# The cGMP Signaling Pathway Affects Feeding Behavior in the Necromenic Nematode *Pristionchus pacificus*

**DOI:** 10.1371/journal.pone.0034464

**Published:** 2012-04-26

**Authors:** Silvina M. Kroetz, Jagan Srinivasan, Jonathan Yaghoobian, Paul W. Sternberg, Ray L. Hong

**Affiliations:** 1 Department of Biology, California State University Northridge, Northridge, California, United States of America; 2 Division of Biology, California Institute of Technology, Pasadena, California, United States of America; Brown University, United States of America

## Abstract

**Background:**

The genetic tractability and the species-specific association with beetles make the nematode *Pristionchus pacificus* an exciting emerging model organism for comparative studies in development and behavior. *P. pacificus* differs from *Caenorhabditis elegans* (a bacterial feeder) by its buccal teeth and the lack of pharyngeal grinders, but almost nothing is known about which genes coordinate *P. pacificus* feeding behaviors, such as pharyngeal pumping rate, locomotion, and fat storage.

**Methodology/Principal Findings:**

We analyzed *P. pacificus* pharyngeal pumping rate and locomotion behavior on and off food, as well as on different species of bacteria (*Escherichia coli, Bacillus subtilis,* and *Caulobacter crescentus*). We found that the cGMP-dependent protein kinase G (PKG) Ppa-EGL-4 in *P. pacificus* plays an important role in regulating the pumping rate, mouth form dimorphism, the duration of forward locomotion, and the amount of fat stored in intestine. In addition, Ppa-EGL-4 interacts with Ppa-OBI-1, a recently identified protein involved in chemosensation, to influence feeding and locomotion behavior. We also found that *C. crescentus* NA1000 increased pharyngeal pumping as well as fat storage in *P. pacificus.*

**Conclusions:**

The PKG EGL-4 has conserved functions in regulating feeding behavior in both *C. elegans* and *P. pacificus* nematodes. The Ppa-EGL-4 also has been co-opted during evolution to regulate *P. pacificus* mouth form dimorphism that indirectly affect pharyngeal pumping rate. Specifically, the lack of Ppa-EGL-4 function increases pharyngeal pumping, time spent in forward locomotion, and fat storage, in part as a result of higher food intake. Ppa-OBI-1 functions upstream or parallel to Ppa-EGL-4. The beetle-associated omnivorous *P. pacificus* respond differently to changes in food state and food quality compared to the exclusively bacteriovorous *C. elegans*.

## Introduction

Evolutionary changes in development and behavior take place in the context of ecology. In turn, what and how organisms eat are the most salient aspects of their ecology readily observable under laboratory conditions. Because of its exclusively bacteriovorous diet, relatively simple neuronal architecture, and genetic tractability, the nematode *Caenorhabditis elegans* is an attractive model for the study of how gene expression impinges upon feeding behavior, and how food availability and quality can interact with genotypes [1], [2]. In *C. elegans,* as well as in other free-living nematodes, the most prominent organ involved in feeding is the pharynx. Food intake begins at the buccal cavity and is pumped into two pharyngeal bulbs composed of the anterior bulb (corpus), the isthmus, and the posterior bulb (terminal bulb). Contractions of the pharyngeal muscle groups (pumping) followed posteriorly sweeping relaxation of the muscles in the isthmus (peristalsis) result in food ingestion in *C. elegans* [3].

*P. pacificus* is a necromenic nematode specifically associated with several species of phytophagous beetles around the globe, in particular the Oriental Beetle *Exomala orientalis* in Japan and Northeastern US, as well as the *Cyclocephala* masked chafers in Southern California (RL Hong, unpublished results) [4]–[6]. Free-living *P. pacificus* populations can also be found in the soil and maintained on strict bacterial diets in the laboratory. *P. pacificus* belongs to the Diplogasteridae family of nematodes whose common ancestor with the Rhabditidae family diverged approximately 250–420 million years ago [7]. Unlike the Rhabditid *C. elegans*, Diplogastrids lack pharyngeal grinders in the terminal bulb (posterior pharynx) that help to breakup bacteria but instead have a larger buccal cavity anterior to the pharynx that contain two chitinous teeth. *P. pacificus,* in particular, have phenotypic dimorphism consisting of two mouth forms, one with a narrower buccal cavity called the stenostomatous form, and another with a broader buccal cavity known as the eurystomatous form [8]–[10]. The evolution of teeth in *Pristionchus* nematodes may be advantageous for the necromenic lifestyle in which *Pristionchus* can feed on various food sources such as bacteria, fungi, and other nematodes. All *Pristionchus* species however, can sustain itself on an *E. coli*-only diet in the laboratory.

Despite such divergent feeding physiology and natural ecology, we currently have sparse knowledge of *P. pacificus* feeding behavior. A recent study showed that *P. pacificus* decrease forward bending frequency from on to off food state whereas *C. elegans* increase forward bending frequency when transferred from on to off food [11]. Other free-living nematodes such as *Oscheius myriophila*, *Rhabditella*, and *Pellioditis typica* display similar reactions as *C. elegans* to changes in food state [11]. Only *Panagrellus redivivus* displayed the same feeding behavior as *P. pacificus*. Changes in foraging behavior by speeding up or slowing down locomotion presumably need to be coordinated with changes in food intake. In *C. elegans,* the feeding rate is measured by the rate of pharyngeal pumping– pumping is higher on food than in the absence of food [12]. Thus *P. pacificus* responds to a lack of food by slowing down locomotion, whereas *C. elegans* increase locomotion but reduce pharyngeal pumping when food is removed. This study will address how *P. pacificus* adjust food pumping rate in response to food availability and food quality.

In *C. elegans,* a pleiotropic gene known to integrate environmental perception, behavior, and growth is *Cel-egl-4*. Cel-EGL-4 is a highly conserved cGMP dependent protein kinase G (PKG) important for a variety of food-seeking behaviors, such as pharyngeal pumping, chemosensation, and locomotion [13]–[16]. PKGs in other invertebrates such as the fruit fly *Drosophila* and the honeybee *Apis mellifera* also play prominent roles in regulating foraging and a polymorphism in PKG is maintained in wild *Drosophila* populations [17], [18]. Past studies in *P. pacificus* demonstrated that differences in *Ppa-egl-4* expression level is involved in the natural polymorphism for an insect sex pheromone attraction between two *P. pacificus* strains [16]. In this study, we found that the *Ppa-egl-4(tu374)* null allele animals are predominantly stenostomatous in mouth form, compared to the ∼2∶1 stenostomatous:eurystomatous ratio found in the wild-type PS312 population [10]. Since EGL-4 function is conserved across phyla and can generate phenotypic changes both at the macroevolutionary level (from insects to nematodes), as well as at the microevolutionary level (within *Drosophila* and *P. pacificus* populations), we sought to address in this study the hypothesis that EGL-4 can also be responsible for coordinating genetic changes in feeding behavior at an intermediate evolutionary scale by comparing the feeding behaviors of *P. pacificus* and *C. elegans* nematodes.

## Results

### *P. pacificus* Pharyngeal Pumping Rate Changes in Response to Food Availability and Food Type

**Figure 1:**
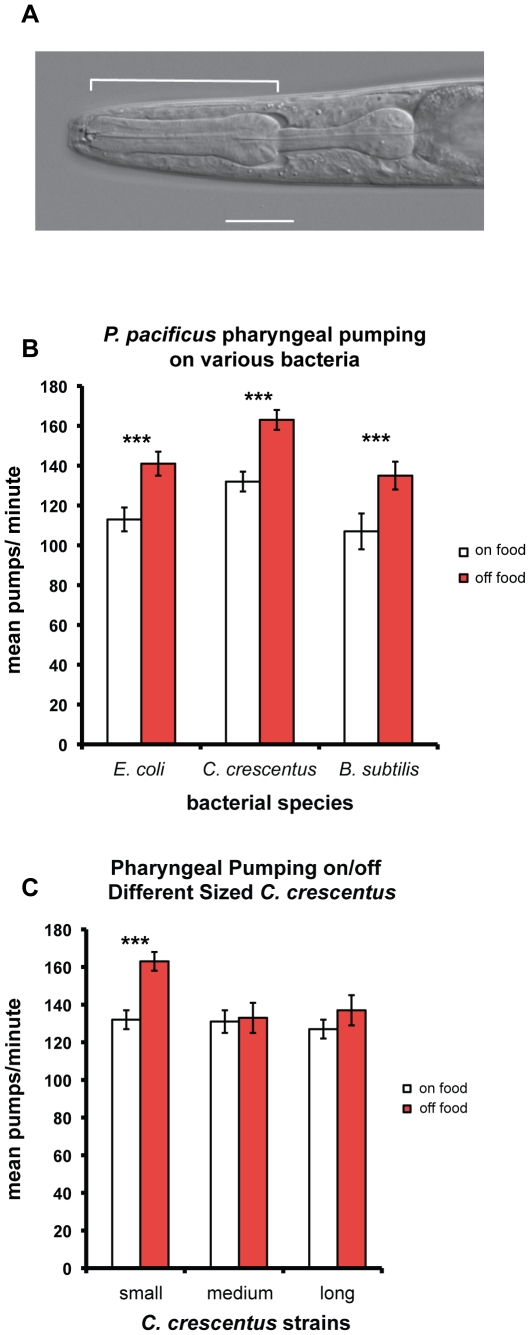
*P. pacificus* pharynx and pharyngeal pumping rate on various bacteria species and strains (mean±SEM). (A) Wild-type PS312 pharynx (Nomarski DIC). Pharyngeal pumping is visible as contractions of the muscles of the corpus (bracketed, anterior is right). The isthmus and the terminal bulb are visible on the right. The scale bar represents 20 µm. (B) Pumping rates of *P. pacificus* wild-type PS312 cultured on *E. coli* OP50 (n = 25–36), *C. crescentus* NA1000 (n = 35–38), or *B. subtilis* PY79 (n = 20–31) were measured on food or off food. The rate difference between on/off food states on each bacterial strain is significant. (C) Pharyngeal pumping rate of *P. pacificus* PS312 cultured on different sizes of *C. crescentus* strains. Small wild-type NA1000 (n = 35–38), medium SM921 (n = 13–20), large LS2195 (n = 18–21) strains were used as the food source. Only the rate difference between on/off food states on the smallest strain (wild-type NA1000) is significant. (Two tailed *t*-test, *P*<0.001).

Both *P. pacificus* and *C. elegans* live solely on *E. coli* OP50 under normal laboratory conditions. To investigate whether food types can alter pumping rate, we cultured *P. pacificus* PS312 for more than six generations on two other bacterial species found in soil− the gram-negative *Caulobacter crescentus* NA1000 found in freshwater and neutral soil [20] and the gram-positive *Bacillus subtilis* PY79 [21]. Wild-type PS312 cultured on *E. coli* OP50 showed ∼25% increased pharyngeal pumping rate (pumps per minute of the corpus, ppm (Fig. 1A) when removed from food for at least 5 minutes. Similarly, PS312 cultured on *C. crescentus* and *B. subtilis* showed increased ppm when removed from food by ∼23% and ∼26%, respectively (Fig. 1B). However, we were surprised to find the magnitude of increase in pumping to be the same, although these 3 bacterial species differ in size (*C. crescentus* = *E. coli*<*B. subtilis*) and growth rate (*C. crescentus*<*B. subtilis<E. coli*) [3], [22], [23]. Interestingly, *P. pacificus* pumps more rapidly both on and off *C. crescentus* than on and off *E. coli* and the larger *B. subtilis.* This observation suggests that food size alone does not influence the pumping rate.

Our finding that *P. pacificus* increases pharyngeal pumping soon after food deprivation, but that the magnitude of this initial starvation response does not differ on the three diverse species of bacteria we tested, led us to further ask if changes in the size and growth rate within a single bacteria species can affect the increase in pumping rate off food. We took advantage of the availability of *C. crescentus* cell cycle mutants by feeding *P. pacificus* on two other strains of *C. crescentus* with defects in the DNA replication regulator (*ctrA*) that affect the cell length as well as the growth rate. SM921 cells (medium) are on average twice as long as the wild-type NA1000 (small) while LS2195 cells (large) are more than twice as long as NA1000 (S. Murray, unpublished results) [24]. We found that nematodes grown on the longer, slower dividing *C. crescentus* strains displayed no increase in pumping rate off food, resulting in the same rate as on food (∼130 ppm) (Fig. 1C). Hence, increasing the *C. crescentus* cell length or reducing its growth rate or both seemed to abolish the immediate increase in pumping rate off food, although the pumping rate on food remained unchanged. This lack of response may be due to the longer time it takes to sense the removal of the longer mutant bacteria, or a complete elimination of the response to food removal. It remains unclear how the longer bacteria size or slower metabolism of the mutant strains causes this delayed “panic” effect. It makes sense that nematodes pump faster when facing possible starvation, but it will be more interesting to understand in the future why certain food quality can suppress this immediate reaction to food removal.

### The cGMP Signaling Pathway is Important for Controlling Pharyngeal Pumping Rate in *P. pacificus*

*P. pacificus* is a free-living nematode capable of eating non-bacterial food such as fungi and other nematodes [9], [10]. Previous comparative studies highlighted that one major difference between *P. pacificus* and *C. elegans* foraging behavior is the decrease in locomotion from on to off food state [11]. Hence, we examined to see if there are other differences in feeding behavior in *P. pacificus,* such as food intake rate. In *C. elegans* wild-type N2, the pharyngeal pumping rate is measured by counting the pumping movements of the grinder in the terminal bulb of the pharynx, and pumping decreases initially when transferred from a well-fed state on food (*e.g. E. coli* OP50) to an off food state without any bacteria (<1 hour) [12] Because *P. pacificus* do not pump in the terminal bulb but instead pump only in the corpus (anterior bulb), we measured pumping rate by visually counting the contractions of the corpus. We found that, unlike *C. elegans* N2, *P. pacificus* wild-type PS312 showed increased pumping rate when off food compared to on food, although both rates are much lower than the pumping rate of *C. elegans* (>200 ppm).

To determine which genes affect pumping rate, we first measured the pumping rate of the *Ppa-egl-4*(*tu374)* loss-of-function null allele [16]. *P. pacificus* EGL-4 is the ortholog of the *C. elegans* cGMP dependent protein kinase G (PKG) known to regulate pharyngeal pumping, chemosensation, and locomotion [13]–[15], [25], [38]. The *Cel-egl-4* loss-of-function mutants lack feeding quiescence after fasting and pumps faster than wild-type N2 on food (pers. comm. van Buskirk) [3]. We found that the loss of Ppa-EGL-4 function also greatly increased pumping off food compared to wild-type PS312, although pumping on food remained the same between wild-type and *Ppa-egl-4.* The *Ppa-egl-4* locus has also been shown to be involved in the natural polymorphism of chemoattraction towards the lepidopteran sex pheromone *E*-11-tetradecenyl acetate (E-TDA) [16]. *P. pacificus* Washington (PS1843) is strongly attracted to E-TDA whereas the California (PS312) reference strain is completely insensitive to E-TDA. However, a brief one-hour soaking treatment of PS312 with the stable, cell permeable 8-bromo-cGMP can increase *Ppa-egl-4* transcript level and induce PS312 chemoattraction to E-TDA [16]. The same exogenous cGMP treatment can also induce PS312 chemoattraction to the sex pheromone of the Oriental Beetle, *Z*-7-tetradece-2-one (Z-TDO), but this cGMP-dependent ZTDO attraction does not wholly depend on *Ppa-egl-4* [16].

**Figure 2:**
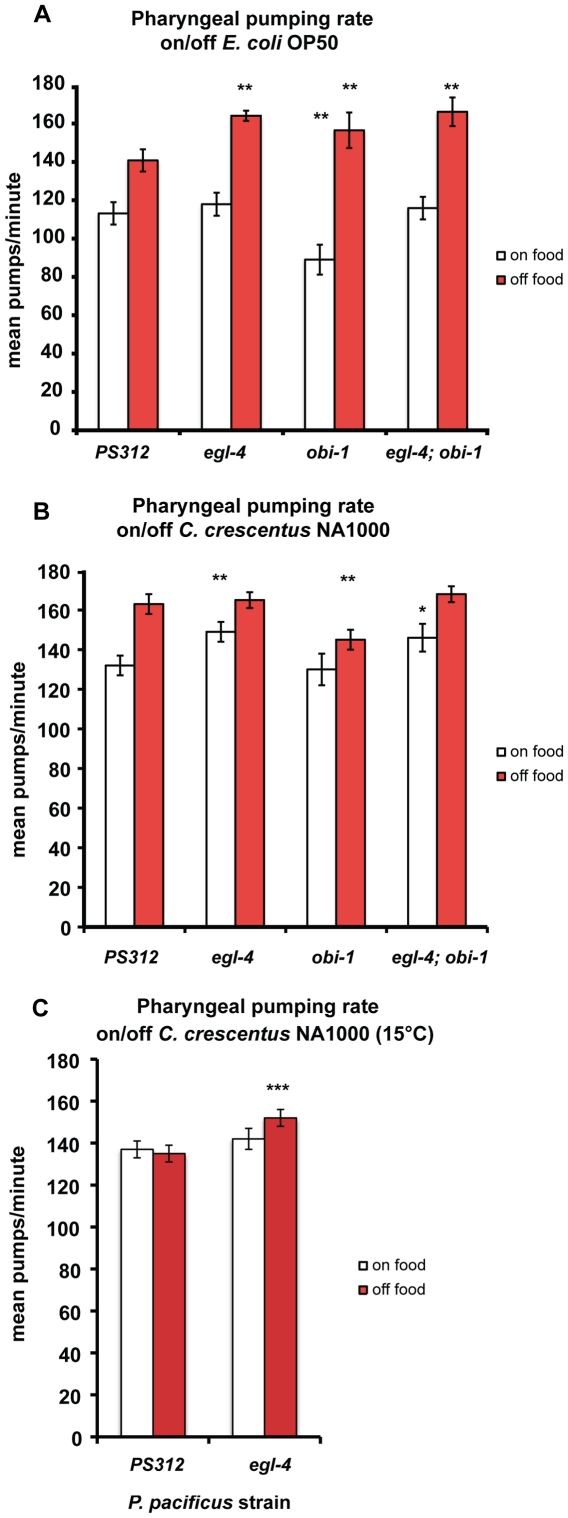
Pharyngeal pumping rate of *P. pacificus* mutants on/off OP50 E. coli and NA1000 *C. crescentus* (mean±SEM). (A) Pumping on/off OP50 *E. coli.* Wild-type PS312 (n = 25–36), *Ppa-egl-4(tu374)* (n = 17–32), *Ppa-obi-1(tu404)* (n = 13–20), *Ppa-egl-4; Ppa-obi-1* (n = 15). The difference in pumping between on/off food states for each nematode strain is significant (not indicated; two tailed *t*-test, *P<*0.001). Pumping rate on food is significantly slower in *Ppa-obi-1(tu404)* compared to the other 3 genotypes, whereas pumping rate off food is significantly faster in all mutants compared to wild-type PS312 (Dunnett’s Multiple comparisons test, *P*<0.01). (B) Pumping on/off NA1000 *C. crescentus*. PS312 (n = 35–38), *Ppa-egl-4* (n = 16–18), *Ppa-obi-1(tu404)* (n = 14–18), *Ppa-egl-4; Ppa-obi-1* (n = 14–18). The difference between on/off food states for each nematode strain is significant (two tailed *t*-test, *P*<0.001). Pumping rate off food is significantly slower in *Ppa-obi-1(tu404)* compared to the other 3 genotypes, whereas *Ppa-egl-4* and *Ppa-egl-4; Ppa-obi-1* pump faster than wild-type (Dunnett’s Multiple comparisons test, *P*<*0.05,**0.01). (C) Pumping on/off on *C. crescentus* NA1000 at 15°C. PS312 (n = 26-32), *Ppa-egl-4* (n = 22–24). The rate difference between on/off food states for *Ppa-egl-4* is significant (two tailed *t*-test, *P*<0.001). *Ppa-egl-4* pumps faster than wild-type on *C. crescentus* (Dunnett’s Multiple comparisons test, *P*<***0.001).

Next, in an effort to find other factors that mediate the exogenous cGMP-dependent attraction to Z-TDO in *P. pacificus* PS312, we isolated an Oriental Beetle pheromone Insensitive allele, *Ppa-obi-1(tu404),* that no longer chemotax to Z-TDO after cGMP treatment (RL Hong, unpublished data). We wondered if the loss of *Ppa-obi-1,* in addition to altering chemosensation, could also affect feeding behavior. We found that *Ppa-obi-1* animals had a lower pumping rate on *E. coli* compared to wild-type, an effect that is suppressed by the loss of EGL-4 function. However, *Ppa-obi-1*, *Ppa-egl-4*, and *Ppa-egl-4; Ppa-obi-1* double mutants all displayed a significantly higher pumping rate off food than wild-type PS312, although this increase is more variable in *Ppa-obi-1* animals. This result suggests that Ppa-OBI-1 promotes pumping on food but negatively regulates Ppa-EGL-4 to control pumping rate off food. Thus, the loss of *Ppa-obi-1* resulted in decreased pumping rate on food while the loss of *Ppa-egl-4* and/or *Ppa-obi-1* resulted in increased pumping rate off food (Fig. 2A). The mutant phenotypes also imply that pharyngeal pumping on and off food are regulated differently by the cGMP pathway.

Since *P. pacificus* cultured on *C. crescentus* displayed increased pumping on and off food, we asked if Ppa-EGL-4, which regulates pumping rate both on and off *E. coli*, also regulates this process on *C. crescentus*. We found that both *Ppa-egl-4* and *Ppa-egl-4; Ppa-obi-1* double mutants on *C. crescentus* NA1000 showed increased pumping on food, but not off food (Fig. 2B). The loss of *Ppa-obi-1* did not affect pumping rate on *C. crescentus*. In contrast, *Ppa-obi-1* off food had decreased pumping. The reduction in Ppa-EGL-4 or Ppa-OBI-1 function also reduced the magnitude of pumping increase from on to off food (11–15% increase in the mutants compared to 23% increase in the wild-type). More interestingly, the off *C. crescentus* pumping rate is the same between *Ppa-egl-4* and wild-type, in contrast to the faster pumping rate of *Ppa-egl-4* on *E. coli*. One explanation could be that ∼160 ppm is near the maximum physiological limit for pharyngeal pumping rate in *P. pacificus*. To address this possibility, we cultured nematodes at 15°C and found that the lower temperature decreased the pumping rate of both wild-type and *Ppa-egl-4* animals. However, *Ppa-egl-4* off *C. crescentus* displayed only a modest increase in pumping compared to wild-type at 15°C, but not when compared to the magnitude of increase between on to off food animals grown at 20°C (Figs. 2A and 2C). Therefore, though it is possible that the lack of a stronger increase in pumping in the mutants off *C. crescentus* is due in part to the inability of the muscles to pump faster at 20°C, the degree of difference on and off *C. crescentus* in *Ppa-egl-4* is fixedly lower than that of *Ppa-egl-4* on and off *E. coli*. This finding also suggests that Ppa-EGL-4 has two roles: Ppa-EGL-4 controls pumping rate off food regardless of food type but controls pumping rate in a food-specific manner, such as on *C. crescentus,* but not on *E. coli.* In contrast, *Ppa-obi-1* controls pumping rate both on and off food for *E. coli*, but only off food for *C. crescentus*.

### Ppa-EGL-4 Affects Mouth Form Dimorphism and Pumping Rate in *P. pacificus*

Unlike *C. elegans*, distinct genetically homogeneous *P. pacificus* populations are each composed of two distinct subpopulations with different mouth forms [8]–[10]. The ratio of stenostomatous to eurystomatous mouth forms in the wild-type PS312 population (51±17% stenostomatous) can be altered by passage through the dauer larvae stage (100% stenostomatous) [10]. Interestingly, we found that *Ppa-egl-4(tu374)* is a genetic mutant that distorts the mouth form ratio towards a high percentage of the stenostomatous morph (87±11%). Because the stenostomatous mouth form has a narrower, longer buccal cavity, more stenostomatous animals in the *Ppa-egl-4* population contribute to the increase in pharyngeal pumping rate in *Ppa-egl-4* animals.

**Figure 3:**
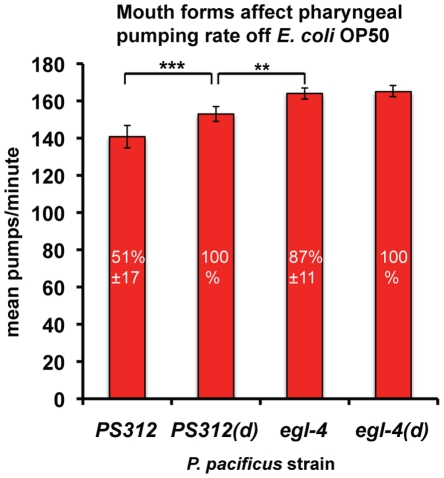
Mouth form affect pharyngeal pumping rate off OP50 *E. coli*. The proportion of young adult hermaphrodites with stenostomatous mouth form changes in the population with dauer passage (d) (percentage of stenostomatous indicated inside the bars). The pumping rate of stenostomatous dauer passaged wild-type is intermediate between not-dauer passaged wild-type and the mostly stenostomatous *Ppa-egl-4* mutants, regardless of dauer passage (Dunnett’s Multiple comparisons test, *P*<**0.01,***0.001).

To determine if the loss of *Ppa-egl-4* affected directly the pumping rate via pharyngeal muscles or indirectly via distortions in mouth form ratio in the population, we sought to measure the pumping rate of a population composed of only a single mouth morph. To do this, we measured dauer passaged wild-type and *Ppa-egl-4* animals cultured on OP50 because they are nearly 100% stenostomatous (Fig 3). We found that the predominantly stenostomatous wild-type population pumped faster than the not-dauer passaged animals, although still not as fast as the *Ppa-egl-4* mutants, regardless of dauer passage. Therefore, the increased pumping rate in the *Ppa-egl-4* mutants is due both to a direct physiological effect on pharyngeal neurons as well as an indirect developmental effect of increasing the likelihood of stenostomatous mouth formation.

### *Ppa-egl-4* Mutants Show Increased Roaming and Forward Velocity

**Figure 4:**
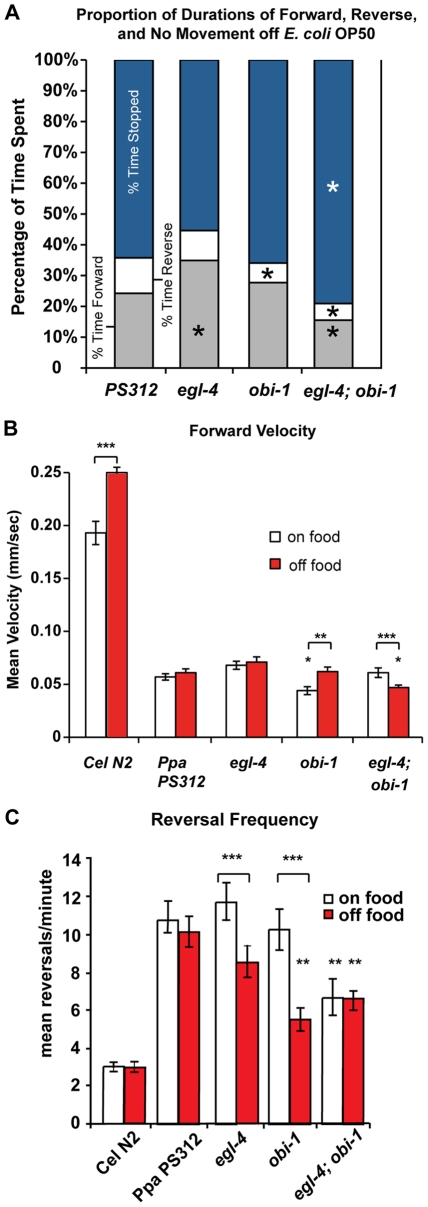
Locomotion of *P. pacificus* mutants (mean±SEM). (A) Proportion of durations *P. pacificus* mutants spent off food (*E. coli* OP50) moving forward, moving backwards, and not moving. PS312 (n = 7), *Ppa-egl-4* (n = 20), *Ppa-obi-1* (n = 18), *Ppa-egl-4;Ppa-obi-1* (n = 9). The percentage time spent is significantly different between wild-type and the mutant strains (Dunnett’s Multiple Comparisons Test *P<0.05). (B) Forward velocities of *C. elegans* N2 and *P. pacificus* strains measured on or off food. Velocity is in millimeters per second cultured on *E. coli* OP50. *C. elegans* wild-type N2 (n = 31–94), *P. pacificus* wild-type PS312 (n = 16–25), *Ppa-egl-4* (n = 16–24), *Ppa-obi-1* (n = 20–22), *Ppa-egl-4; Ppa-obi-1* (n = 11–27). The differences between on and off food states for *C. elegans* N2, *Ppa-obi-1* and *Ppa-egl-4; Ppa-obi-1* are significant (two-tailed *t*-test, *P*<***0.001, <**0.01). Forward velocities are significantly different from wild-type on food for *Ppa-obi-1* as well as off food for *Ppa-egl-4; Ppa-obi-1* (Dunnett’s Multiple Comparisons test, *P*<0.05). (C) Reversal frequencies of *C. elegans* and *P. pacificus* mutants. Reversals frequencies of nematodes cultured on *E. coli* OP50 were measured on or off food. *C. elegans* wild-type N2 (n = 25–51), *P. pacificus* wild-type PS312 (n = 16–25), *Ppa-egl-4* (n = 16–24), *Ppa-obi-1* (n = 20–22), and *Ppa-egl-4; Ppa-obi-1* (n = 11–27). Reversal frequencies between on and off food states for *Ppa-egl-4* and *Ppa-obi-1* strains are significantly different (two-tailed *t*-test, *P*<***0.001). Reversal frequencies on food are significantly different between wild-type and *Ppa-egl-4*; *Ppa-obi-1*. Reversal frequencies off food are significantly different between wild-type and *Ppa-obi-1* as well as *Ppa-egl-4*; *Ppa-obi-1* (Dunnet’s Multiple Comparisons test, *P*<0.01).

Nematodes on solid media can be observed as moving forwards, backwards, or not at all (stopped for at least 4 seconds). Unlike *C. elegans,* which increases locomotion (body bends/minute) when transferred from on to off food, *P. pacificus* decreases locomotion when deprived of food from a well-fed state [11]. Because the pharynx pumps constantly, we wished to determine if the increase in pumping rate of *Ppa-egl-4* off food is coupled with alterations in foraging behavior. The PKG in other invertebrates such as Drosophila and honey bee also play a key role in food-seeking behavior [17], [18]. In *C. elegans*, *Cel-egl-4* loss-of-function mutants show increased roaming behavior on food compared to wild-type, as exhibited by a more time spent on forward locomotion [14], [26], while *Cel-egl-4* gain-of-function mutants displayed increase in dwelling, as signified by more stops and reversals [25]. We compared the foraging behaviors of the various *P. pacificus* feeding mutants in the absence of food (*E. coli* OP50). The loss-of-function *Ppa-egl-4(tu374)* null animals spent more percentage of time off food moving forward (*P*<0.05, Fig. 4A). In contrast, the *Ppa-obi-1* mutant showed decreased reverse durations compared to wild-type (*P*<0.01), although the durations moving forward were not significantly different than wild-type. Furthermore, *Ppa-egl-4; Ppa-obi-1* animals spent more of their time not moving compared to the wild-type or single mutants (*P*<0.05), and also differs in duration of forward and reverse movements compared to wild-type (*P*<0.01). Thus, the loss-of-function *Ppa-egl-4* mutants in *P. pacificus,* as in the case of *C. elegans,* displayed an increased proportion of forward movements, while the *Ppa-obi-1* mutation completely reversed the *Ppa-egl-4* roaming phenotype. However, the *Ppa-egl-4; Ppa-obi-1* double mutant animals displayed forward and stopped durations not observed in the single mutants while the reduced reversal duration of *Ppa-obi-1* appears to be epistatic to *Ppa-egl-4*.

In addition to differences in the percentage of time spent on certain movements, we examined whether *P. pacificus* and *C. elegans* differ fundamentally in speed and reaction to change in food state by measuring mean forward velocity on and off food. We found that not only is *P. pacificus* significantly slower than *C. elegans* by 4–5 fold both on and off food, *P. pacificus* also does not display a dramatic increase in velocity when food is removed from well-fed individuals as is the case in *C. elegans* (Fig. 4B). Furthermore, we found differences between wild-type *P. pacificus* and mutants in the cGMP pathway. Specifically, although *Ppa-egl-4* mutant animals spent more time moving forward, they do not move faster on or off food compared to wild-type. *Ppa-obi-1* however, is slower than wild-type on food. In contrast, the *Ppa-egl-4; Ppa-obi-1* double mutants have significantly reduced forward velocity off food compared to the wild-type or *Ppa-egl-4* mutants alone. We speculate that Ppa-OBI-1 acts upstream of Ppa-EGL-4 to positively regulate forward velocity in the absence of food, although in a more complex manner than for pharyngeal pumping. Interestingly, the *Ppa-obi-1* mutant exhibited a similar degree of strong increase in velocity from on to off food (40% faster off food) observed in the *C. elegans* wild-type (30% faster off food).

We found equally profound differences between *P. pacificus* and *C. elegans* when we focused on the reversal frequency. Whereas *C. elegans* wild-type moved ∼5x faster forward, *P. pacificus* wild-type reversed almost 4x more frequently than *C. elegans* (Fig. 4C). Given its slow forward velocity, *P. pacificus* may instead increase reversal frequency to enhance coverage of the local area rather than increase its foraging range when confronted with possible starvation. The reversal frequency of *Ppa-egl-4* and *Ppa-obi-1* were less on food than off food in contrast with wild-type and *Ppa-egl-4; Ppa-obi-1* double mutants. In the case of *Ppa-egl-4; Ppa-obi-1* double mutants however, reversal frequency was equally low on and off food. The decreases in reversal frequency coincided with increases in pumping rate off food in *Ppa-egl-4, Ppa-obi-1,* and *Ppa-egl-4; Ppa-obi-1* double mutants. This result suggests that when *P. pacificus* lacks food, Ppa-EGL-4 functions to coordinate foraging patterns by regulating the proportion of time moving forward, reversal frequency, and the rate of pharyngeal pumping.

### *C. crescentus* Increases *P. pacificus* Fat Content

**Figure 5:**
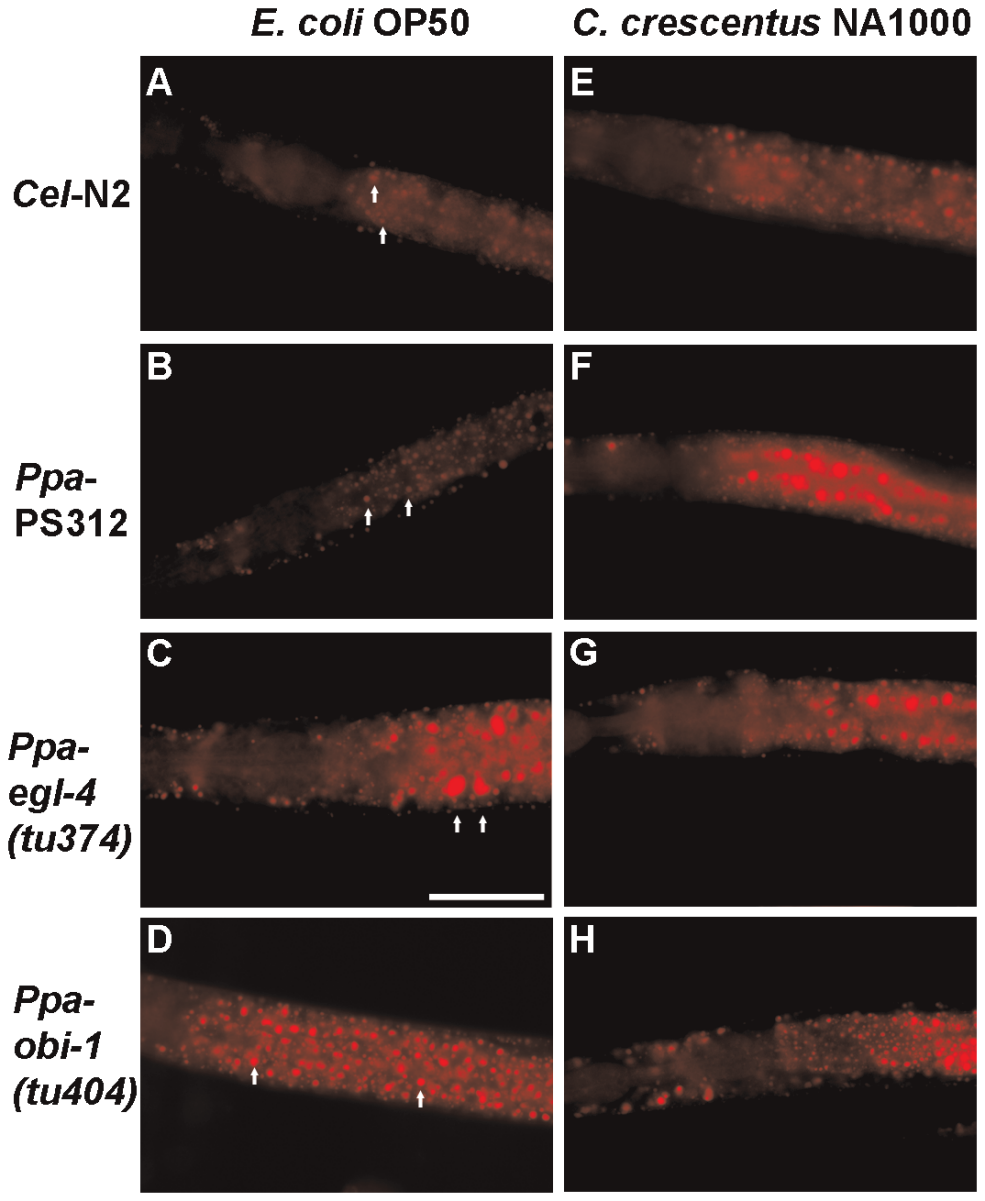
Representative Nile Red staining of fixed nematodes. The diameters of the two largest stained lipid droplets in the anterior intestine were measured for fat quantification and are indicated with arrows for the OP50-fed worms as examples. Anterior is to the left and the scale bar for (C) represents 25 µm. Images on the left column (A-D) show nematodes cultured on *E. coli* OP50 while images (E-H) on right column show nematodes cultured on *C. crescentus* NA1000. Nematodes were fixed in paraformaldehyde and stained with Nile Red.

**Figure 6:**
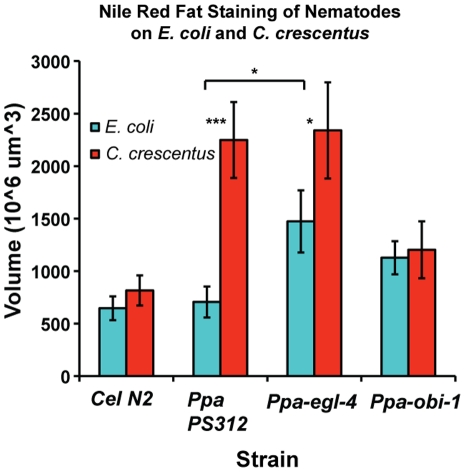
Comparisons of fat storage in the intestines of fixed nematodes. The diameters of the two largest stained bodies per animal in the anterior intestine between the end of the pharynx and the invaginating vulva of L4 or J4 stage animals were measured to calculate the average volume based on spherical geometry (mean±SEM). Comparable gain and exposure settings were used. *C. elegans* wild-type N2 (n = 38–52), *P. pacificus* wild-type PS312 (n = 34–45), *Ppa-egl-4* (n = 37–55), *Ppa-obi-1* (n = 36–52). Fat storage on *E. coli* OP50 was higher in *Ppa-egl-4* than in wild-type PS312. Fat storage in both PS312 and *Ppa-egl-4* was higher when cultured on *E. coli* than on *C. crescentus* (***P<0.001; *P<0.05). Fat storage was higher on *C. crescentus* in both PS312 and *Ppa-egl-4* than in *C. elegans* N2 and *Ppa-obi-1* (not indicated; *P*<0.01). (Tukey-Kramer Multiple comparisons test, *P*<0.05).

A previous study in *C. elegans* by Shtonda and Avery (2006) demonstrated that the presence of high quality food correlated with changes in locomotion behavior [40]. We hypothesized that the observed difference in pharyngeal pumping behavior due to differences in food or genotype would also influence the nutritional status of the nematodes. Wild-type *P. pacificus* pumped food more rapidly on and off *C. crescentus* NA1000 than on and off *E. coli* OP50. We therefore asked if the increase in pumping on *C. crescentus* has consequences in fat accumulation because it is more nutritious. We utilized the Nile Red dye to detect triacylglyceride (TAG) content in the intestines of fixed animals [27]. The size of stained lipid bodies in the anterior intestine of *C. elegans* N2 and *P. pacificus* PS312 were similar in size when fed on *E. coli* OP50, but increased dramatically when *P pacificus* strains were cultured on *C. crescentus* (Fig. 5). This supports the notion that the faster pumping on *C. crescentus* may be driven by preference for bacteria that provide higher fat content, rather than a reaction to lower density of food per pump. Interestingly, *C. elegans* cultured on *C. crescentus* did not show a significant increase in fat storage compared to those cultured on OP50 and thus may reflect a very different metabolism from *P. pacificus* (Fig. 6). Next, we investigated whether the higher pumping rate in *Ppa-egl-4(tu374)* on *C. crescentus* would result in even higher fat accumulation. To our surprise, the fat storage level was unchanged compared to the wild-type on *C. crescentus*, but the fat level in *Ppa-egl-4* cultured on OP50 was found to be higher than in wild-type PS312 (*P*<0.05). This result is in contrast to the previous finding that a *Cel-egl-4* gain-of-function allele did not accumulate more fat using the same Nile Red staining method [27]. Unlike the wild-type, *Ppa-obi-1* did not accumulate more fat when cultured on *C. crescentus* (*P*<0.001, Tukey-Kramer). Thus, *C. elegans* N2 did not seem to accumulate fat differently on *E. coli* and *C. crescentus,* whereas *P. pacificus* PS312 accumulated almost 3x more fat on *C. crescentus* compared to on *E. coli*. The higher fat accumulation also correlated with the faster pharyngeal pumping on *C. crescentus* compared to on *E. coli* in wild-type *P. pacificus*. However, this correlation between pharyngeal pumping rate and fat storage level did not hold for *Ppa-egl-4* and *Ppa-obi-1* mutants, perhaps due to the distinct but overlapping regulatory circuits for pharyngeal pumping on and off food. Alterations in these two genes can affect the magnitude of this fat storage difference between *E. coli* and *C. crescentus* diets. *Ppa-egl-4(tu374)* specifically increases the fat level on OP50 compared to wild-type, whereas *Ppa-obi-1* reduces the fat level on *C. crescentus* compared to wild-type.

## Discussion

*P. pacificus* is a necromenic nematode specifically associated with several species of beetles, whereas *C. elegans* is primarily found in vegetable composts [5], [6], [19], [28]. In the course of evolution, their contrasting ecologies produced stark differences in feeding behavior as evident by their feeding organs. *P. pacificus* lack pharyngeal grinders but have teeth and show predatory behavior towards *C. elegans* and other nematodes (RJ Sommer, personal communication) [10]. The ability of *P. pacificus* to catch and kill moving prey, coupled with their ability to proliferate in the laboratory solely on bacteria, suggest *P. pacificus* has a flexible diet and potentially complex feeding strategies in nature. Our present study interrogates the relationship between *P. pacificus* genotypes and their responses to food states and food types.

### Comparison of EGL-4 Functions

**Figure 7:**
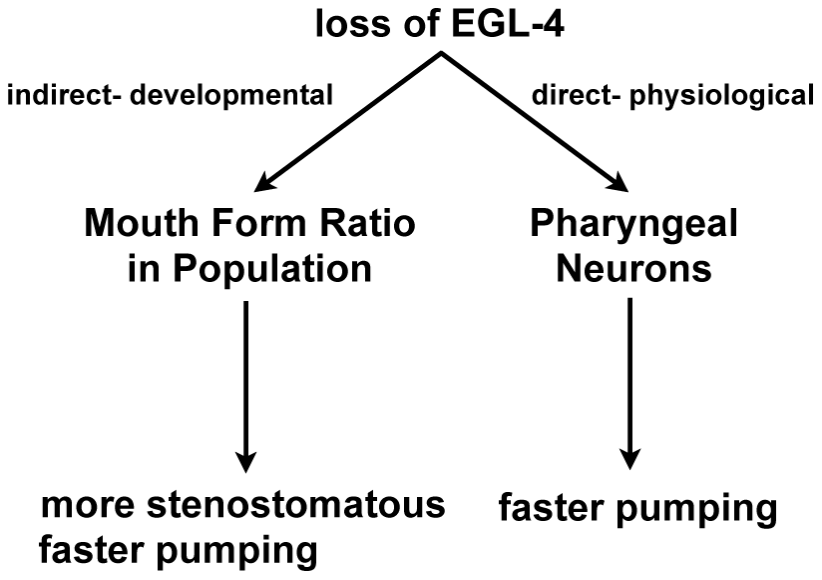
Model for direct and indirect Ppa-EGL-4 regulation of pharyngeal pumping rate on *E. coli* OP50. The loss of Ppa-EGL-4 increases pumping rate directly by regulating pharyngeal neurons, as well as indirectly by increasing the number of animals with the narrower stenostomatous mouth form in the population.

The PKG EGL-4 has conserved functions in regulating feeding behavior in both *C. elegans* and *P. pacificus*. Specifically, the *Ppa-egl-4* mutants increased their rate of pharyngeal pumping, along with increased duration roaming off food through increased proportion of time moving forward. Interestingly, the *Ppa-egl-4* mutants did not pump faster on OP50 but only off of OP50. One interpretation is that animals lacking Ppa-EGL-4 are more sensitive to the removal of food. This sensitivity may also depend on food metabolism, as shown by the increase in fat storage of *Ppa-egl-4* grown on OP50. *P. pacificus* pumped faster when food is more nutritious (on *C. crescentus*), or when the higher fat storage of the *Ppa-egl-4* mutant animals demands even more food intake from less nutritious food (on *E. coli*). PS312 cultured on *C. crescentus* also seemed to induce more fat accumulation as demonstrated by the animals’ increased pharyngeal pumping and fat storage. However, the nutritional content of *C. crescentus* NA1000 may be significantly different from *E. coli* OP50. Although *Ppa-egl-4* on *C. crescentus* also showed increased pumping on food, *Ppa-egl-4* did not show further increase in fat content, perhaps because the larger size of fat droplets in wild-type *P. pacificus* cultured on *C. crescentus* is already close to the physiological limit. We observed that *Ppa-obi-1*, a chemosensory mutant that does not respond to exogenous cGMP, showed *egl-4*-dependent phenotypes in pharyngeal pumping. This suggests that Ppa-OBI-1 acts upstream to negatively regulate Ppa-EGL-4 in feeding behavior (Fig 7).

In addition to conserved physiological functions, our findings also indicate that Ppa-EGL-4 has also been co-opted to regulate the mouth form dimorphism that is found in the *Pristionchus* genus. We found that wild-type PS312 nematodes are 51±17% stenostomatous, while *Ppa-egl-4(tu374)* nematodes are 87±11% stenostomatous. However, it is not clear if Ppa-EGL-4 normally represses stenostomatous development or promotes eurystomatous development. The development of the mouth form is phenotypically plastic within a single genotype and is influenced by environmental signals, such as starvation [10]. Passage through the dauer larvae, another phenotypically plastic trait induced by starvation, greatly increases the proportion of stenostomatous animals in the population. The stenostomatous mouth form is partly characterized by a longer, narrower buccal cavity that can restrict passage of food particles, hence the mostly stenostomatous *Ppa-egl-4* animals may pump faster to compensate for receiving less food per pump. To discriminate between the direct regulation of pharyngeal neurons versus the indirect developmental effect of mouth form dimorphism by Ppa-EGL-4, we compared dauer passaged wild-type and *Ppa-egl-4* mutants (both ∼100% stenostomatous). Our finding that dauer passaged wild-type pumps faster than not-dauer passaged wild-type (with less stenostomatous) but still slower than *Ppa-egl-4,* regardless of dauer passage, suggests both direct and indirect effect of Ppa-EGL-4 on pharyngeal pumping (Figs. 3 and 7). Therefore, Ppa-EGL-4 regulates feeding behavior by integrating environmental signals with development and physiology. In the wild, it may be advantageous for starvation induced *P. pacificus* dauer juveniles that recovery on limited food to have more individuals with stenostomatous mouth forms that pump faster when subsequently confronted with low food patches.

The role of PKG in regulating food acquisition behaviors is conserved across large evolutionary distances, suggesting the high connectivity of PKG to various cellular signals, from nematodes to humans [29], [30]. However, how PKG functions diverge with respect to specific roles in feeding and metabolism has not been investigated at a shorter evolutionary scale, *i.e.* between members of different taxonomic families such as between the Rhabditidae and Diplogasteridae. Table 1 summarizes and compares the various functions of Ppa-EGL-4 in *P. pacificus* and *C. elegans* based on genetic analyses. Interestingly, although PKG regulate locomotion and pharyngeal pumping in both *P. pacificus* and *C. elegans,* these two nematodes species react in opposite manners to food removal. *C. elegans* respond to food removal by moving faster and pumping less, while *P. pacificus* neither change forward velocity nor reversal frequency, and pumps faster when food is gone. One interpretation for these strategies is that *C. elegans* are more “confident” that they will get to a food source soon but refrain from maximum pumping until they find food, while *P. pacificus* are less “confident” that food is nearby so they remain in the same foraging pattern but pump faster to get the most out of the food that remains. Another major difference between the two nematodes is exhibited by EGL-4′s role in controlling body size. Previous studies show that the loss of EGL-4 function increased body size in *C. elegans* but decreased body size in *P. pacificus* [16], [31]. The loss of EGL-4 function in *C. elegans* reduced fat content whereas *Ppa-egl-4(tu374)* mutants showed higher fat content [25]. However, the Nile Red staining on *Cel-egl-4(lf)* has been performed on live worms and recent work demonstrated that staining on fixed worms more accurately reflects fat content [27]. Nile Red staining on fixed gain-of-function allele *Cel-egl-4(ad450)* specimen did not differ from wild-type N2 in fat storage. The observed phenotypic differences between *P. pacificus* and *C. elegans egl-4* loss-of-function mutants may be due to changes in EGL-4 transcriptional targets [32] or the diverged functions between the two known PKGs in their genomes or a combination of both. Both the *C. elegans* and the *P. pacificus* genomes encode another transcribed PKG paralog (WBGene00015650). Therefore the *egl-4* null alleles in both nematode species likely represent a reduced rather than a complete lack of PKG activities, and thus additional studies on the distributions of functions between the PKG paralogs in both species are needed.

**Table 1:** A comparison of *egl-4* mutant phenotypes in *C. elegans* and *P. pacificus.*

*egl-4* mutation	body length	fat storage	pharyngeal pumping	Forward movement	egg holding	odor sensing	odor adaptation
***Cel-egl-4(lf)***	↑^26,31^	↓^25^	↑^#^	↑^14^	↑^38^	↓^13^	↓^13^
***Ppa-egl-4(lf)***	↓^16^	↑*	↑*	↑*	no affect^16^	↓^16^	↓^16^

In *C. elegans*, the loss-of-function (lf) alleles *n479* and *ks62* have been used in multiple studies; In *P. pacificus*, only the loss-of-function null allele *tu374* have been described. For fat storage measurements, *Cel-egl-4(gf)* and *Ppa-egl-4(lf)* alleles refer to Nile Red staining on fixed animals. *this study; ^#^van Buskirk pers. comm; [13], [14], [16], [25], [26], [31], [38].

### *C. crescentus* Induces Faster Pumping and Fat Storage

Microfauna studies and transmission electron micrographs strongly suggest that *E. coli* OP50 is not effectively digested by *P. pacificus* because intact *E. coli* cells remain in the intestine [33], [34]. This may be because *P. pacificus* lacks grinders to break up bacteria cells and have instead evolved a very different feeding apparatus, the toothed mouth, that is better suited for diverse food sources such as fungal spores and nematodes, in addition to the various bacteria found on beetle carcasses. Nevertheless, because *P. pacificus* can proliferate on the same OP50 diet as *C. elegans,* we tested other bacteria species as food for *P. pacificus* and found that *P. pacificus* pumped more rapidly and stored more fat when cultured on *C. crescentus* than on *E. coli.*

*E. coli* and *C. crescentus* are both gram-negative bacteria. However, *E. coli* has an optimal growth temperature of 37°C commonly found in the intestines of endotherms, while *C. crescentus* is found in freshwater and sub-terrestrial environments with optimal growth temperatures between 23–28°C [20], conditions much closer to where *P. pacificus* and *C. elegans* are found in nature. *C. crescentus* is a model for understanding prokaryotic development and cell cycle regulation because of its dimorphic life cycle involving motile swarmers with flagellum and sessile cells with adhesive stalks [24]. In particular, phospholipids are the primary fatty acids in the cell membranes of bacteria and do not differ significantly among *E. coli* strains, whereas triacylglycerides have been found to differ significantly among OP50, HB101, and HT115 strains of *E. coli* [27]. In addition to cell length, the *C. crescentus* strains with defects in DNA replication used in this study can also differ in metabolism as well as cell cycle rate (S Murray, personal communication). A recent study also indicated that *C. elegans* pump faster on non-pathogenic bacteria than on pathogenic bacteria, especially when selected for fitness during experimental evolution [35], suggesting that *P. pacificus* cultured on *C. crescentus* may find it more nutritious. Our future research will seek to address how different strains of *C. crescentus* may influence pharyngeal pumping rate and metabolism in *P. pacificus*.

### Genetic Interactions between *Ppa-egl-4* and *Ppa-obi-1*

Tables 2 and 3 summarize the changes in pharyngeal pumping and locomotion behavior in wild-type versus *Ppa-egl-4* and *Ppa-obi-*1 mutants. *Ppa-obi-1(tu404)* was isolated from a *P. pacificus* chemoattraction screen for insensitivity to the Oriental Beetle sex pheromone after treatment with exogenous cGMP. Consistent with *Ppa-obi-1′*s function in modulating the cGMP pathway, *Ppa-obi-1(tu404)* lack the enhanced pharyngeal pumping and locomotion phenotypes of *Ppa-egl-4(tu374)*. The *Ppa-obi-1* mutation in the *Ppa-egl-4* background reversed the loss-of-function *Ppa-egl-4* forward roaming phenotype such that the *Ppa-egl-4; Ppa-obi-1* double mutant exhibited more dwelling than wild-type and single mutants. However, the slower pharyngeal pumping and decrease in forward velocity phenotypes of *Ppa-obi-1* on food was masked by *Ppa-egl-4* in the *Ppa-egl-4; Ppa-obi-1* double mutants. The forward velocity off food is slowest in the *Ppa-egl-4; Ppa-obi-1* double mutants, suggesting that Ppa-EGL-4 and Ppa-OBI-1 interactions involved in regulating forward velocity depend on food state. We speculate that based on these epistatic interactions for various feeding phenotypes, Ppa-OBI-1 acts both upstream as well as in parallel to Ppa-EGL-4 in signaling pathways involving other yet-to-be identified factors. Preliminary studies on *Ppa-egl-4* and *Ppa-obi-1* show largely non-overlapping expression patterns, suggesting that these two cGMP signaling components intersect functionally but are expressed in distinct cell types (RL Hong, unpublished results).

**Table 2:** Changes in pharyngeal pumping rate in *Ppa-egl-4* and *Ppa-obi-1* mutants compared to wild-type *P. pacificus.*

Food Type	*egl-4*	*obi-1*	*egl-4;obi-1*
**on ** ***E. coli***	–	↓	–
**off ** ***E. coli***	↑	↑	↑
**on ** ***C. crescentus***	↑	–	↑
**off ** ***C. crescentus***	↑*	↓	–

* at 15°C.

**Table 3:** Changes in locomotion on/off *E. coli* OP50 in *Ppa-egl-4* and *Ppa-obi-1* mutants compared to wild-type *P. pacificus* ***.***

Behavior	*egl-4*	*obi-1*	*egl-4;obi-1*
**% Forward** **(off food)**	↑	–	↓
**% Reverse** **(off food)**	–	↓	↓
**% Stopped** **(off food)**	–	–	↑
**Forward Velocity** **(on food)**	–	↓	–
**Forward Velocity** **(off food)**	–	–	↓
**Reversal Frequency** **(on food)**	–	–	↓
**Reversal Frequency** **(off food)**	–	↓	↓

## Materials and Methods

### Nematode and Bacterial Strains

Nematode strains were cultured between 20–23°C. The wild-type strains of *C. elegans* and *P. pacificus* were N2 Bristol and PS312 California, respectively. *Ppa-egl-4(tu374)* and *Ppa-obi-1(tu404)* were generated in the PS312 background by UV and X-ray mutagenesis and are presumed to be both loss-of-function null mutations (Hong et al 2008 and unpublished results to be described elsewhere). *Ppa-egl-4; Ppa-obi-1* double mutants were obtained by PCR genotyping F_2_ cross progeny with the long body phenotype of *Ppa-obi-1(tu404)* for the 780 bp *Ppa-egl-4(tu374)* deletion [16]. For bacteria, *Escherichia coli* OP50, *Bacillus subtilis* PY79 and *C. crescentus* NA1000 were the reference strains. *E. coli* and *B. subtilis* cultures were inoculated in LB at 37°C while *C. crescentus* cultured were grown in Peptone Yeast Extract (PYE) at 28°C. *C. crescentus* strains with defects in ctrA required for proper DNA replication resulted in the larger cell sizes of SM921 (longer, ctrAP1?)(S. Murray, personal communication) and LS2195 (longest, ctrA405+S) [24]. 150 µl of fresh overnight bacteria inoculates were seeded onto 6-cm culture plates containing Nematode Growth Media (NGM). Nematode cultures were acclimated for at least 6 generations on each bacterial strain before being assessed for pumping rate, locomotion, and fat storage.

### Pharyngeal Pumping Rate

In *C. elegans,* the pharyngeal pumping rate is measured by visually counting the contractions of the “grinder” in the posterior, or terminal pharyngeal bulb. Counts are often observed using video recordings because the pumping rate is too fast to count by eye. Unlike *C. elegans, P. pacificus* does not have grinders and the terminal bulb does not pump, but instead pumps only in the corpus (anterior pharynx). The counts in *P. pacificus* are <200 pumps per minute (ppm) and measured by visually counting the contractions of the corpus using a ZEISS LUMAR stereomicroscope at 80x magnification. Worms from well-fed cultures were placed on assay plates containing either a thin lawn of an overnight culture of the same bacteria (on food) or on plates not containing any food (off food for 5 minutes). For each animal, we counted pumps for five 15-second intervals, with >2 minutes between intervals, and then multiplied its mean by four to derive the mean pumps per minute.

### Mouth Form Dimorphism

To obtain populations composed primarily of the stenostomatous mouth form, we picked wild-type PS312 and *Ppa-egl-4(tu374)* dauer juveniles from 2–3 week old starved culture plates onto OP50 seeded lawns and let them resume development for 3 days at ∼22°C. The mouth forms of young adults of both not-dauer passaged and dauer passaged populations were analyzed at 630x using Nomarski optics and classified as stenostomatous (narrow buccal cavity and flat dorsal tooth) or eurystomatous (wide buccal cavity and barbed dorsal and ventral teeth) according to Bento *et al* 2010 [10]. A total of at least 45 animals from 5 dauer populations in each category were observed for mouth form and animals whose mouth forms cannot be classified were not included in the ratio count.

### Nile Red Fat Staining

We utilized the previously described method of using the lipophilic dye Nile Red (Sigma-Aldrich, N3013) to detect the presence of triacylglycerides and phospholipids in the intestines of fixed animals [27]. Detection of fat storage by Nile Red in live worms is unreliable due to lysosome processing but detection in fixed specimen have been shown to be reproducible and correlated with fat detection by TLC/GC [39], [27]. In brief, nematode cultures were washed twice with M9 buffer and freeze-thawed thrice (liquid nitrogen/30°C water bath) in 500 µl of 0.5% freshly diluted paraformaldehyde in M9 buffer (w/v). The fixed worms were then washed twice with 500 µg/ml of freshly diluted Nile Red solution in PBS buffer and incubated at room temperature for 45–60 minutes. The stained worms were subsequently washed twice with 3x volumes of M9 buffer and placed on 2% Noble agar pads on glass slides for fluorescence microscopy using a Leica DM6000 with the cy5 filter. At least 3 independently grown cultures were analyzed per nematode genotype per food type. Both the size and intensity of stained lipid droplets have been found to correlate with fat levels. However, we found the size of the lipid droplets to be more consistent than the intensity of staining within genotypes. Therefore we measured the diameters of the two largest fluorescent droplets in the medial anterior portion of the intestine (buccal cavity in focus and half way between the pharyngeal-intestinal valve and the invaginating vulva, approximately 50 µm) of L4/J4 stage hermaphrodites cultured on 3 day-old *E. coli* OP50 or *C. crescentus* NA1000 lawns. The mean values of these lipid droplets were expressed as volumes (N×10^6^ µm^3^) using the Leica LAS imaging analysis software.

### Nematode Locomotion and Data Analysis

Worms were tested by automated tracking continuously cultured on *E. coli* OP50, and tested on OP50. We used 10 cm non-seeded NGM plates to test different parameters of locomotion. Worms were placed on assay plates containing either a thin lawn of an overnight culture of *E. coli* OP50 or plates not containing any food [36]. As previously described, 10 cm NGM plates used for recordings were equilibrated to 20°C for 18–20 hours. Approximately one hour before beginning recordings, 600 µl of fresh OP50 overnight culture was spread on each plate to achieve a thin, featureless lawn of food across the entire surface. Excess solution was drawn from the edge with a Pipetman. Food was allowed to dry on the agar surface of a tissue paper-covered plate until the surface exhibited a matte finish (about 45 minutes). L4/J4 hermaphrodites of both *C. elegans* and different mutations of *P. pacificus* were picked to freshly seeded plates 16–20 hours prior to recording. Individual worms were transferred to assay plates and the plate placed in a holder on the microscope stage. After two minutes of recovery, the worm was located and recording begun using an automated worm tracker and image recorder specially designed for studying worm locomotion [36], [37]. Each worm was recorded for five minutes. Data extraction, processing and analysis were done using image processing and analysis software as previously described [36], [37]. From each video recording of 5 minutes, we used the middle 4 minutes, and used the software to derive values for frequency of undulations. All incubations and recordings were done in a constant temperature room at 20°C.

### Statistical Analysis

Means, SEM (error bars), and *P* values for two-tailed *t*-test were performed by Microsoft Excel. Significant *P* values in figures are denoted by: *P<0.05; **P<0.01; ***P<0.001. One-way ANOVA, Tukey-Kramer and Dunnett’s Multiple comparisons post-hoc testing were performed using the InStat statistical software.

## References

[1] Avery L, Bargmann CI, Horvitz HR (1993). The Caenorhabditis elegans unc-31 gene affects multiple nervous system-controlled functions.. Genetics.

[2] Raizen DM, Lee RY, Avery L (1995). Interacting genes required for pharyngeal excitation by motor neuron MC in Caenorhabditis elegans.. Genetics.

[3] Avery L, Shtonda BB (2003). Food transport in the C. elegans pharynx.. J Exp Biol.

[4] Hong RL, Sommer RJ (2006). Pristionchus pacificus: a well-rounded nematode.. Bioessays.

[5] Herrmann M, Mayer WE, Sommer RJ (2006). Nematodes of the genus Pristionchus are closely associated with scarab beetles and the Colorado potato beetle in Western Europe.. Zoology.

[6] Herrmann M, Mayer W, Hong RL, Kienle S, Minasaki R (2007). The nematode Pristionchus pacificus (Nematoda: Diplogastridae) is associated with the Oriental beetle Exomala orientalis (Coleoptera: Scarabaeidae) in Japan.. Zoolog Sci.

[7] Dieterich C, Clifton SW, Schuster LN, Chinwalla A, Delehaunty K (2008). The Pristionchus pacificus genome provides a unique perspective on nematode lifestyle and parasitism.. Nat Genet.

[8] Hirschmann H (1951). Ueber das Vorkommen zweier Mundhoehlentypen bei Diplogaster lheritieri Maupas and Diplogaster biformis n. sp und die Entstehung dieser hermaphroditischen Art aus Diplogaster lheritieri. Zool. Jahrb.. (Syst.).

[9] von Lieven AF, Sudhaus W (2000). Comparative and functional morphology of the buccal cavity of Diplogastrina (Nematoda) and a first outline of the phylogeny of this taxon.. Journal Of Zoological Systematics And Evolutionary Research.

[10] Bento G, Ogawa A, Sommer RJ (2010). Co-option of the hormone-signalling module dafachronic acid-DAF-12 in nematode evolution.. Nature.

[11] Rivard L, Srinivasan J, Stone A, Ochoa S, Sternberg PW (2010). A comparison of experience-dependent locomotory behaviors and biogenic amine neurons in nematode relatives of Caenorhabditis elegans.. BMC Neurosci.

[12] Avery L, Horvitz HR (1990). Effects of starvation and neuroactive drugs on feeding in Caenorhabditis elegans.. J Exp Zool.

[13] L’Etoile ND, Coburn CM, Eastham J, Kistler A, Gallegos G (2002). The cyclic GMP-dependent protein kinase EGL-4 regulates olfactory adaptation in C. elegans.. Neuron.

[14] Fujiwara M, Sengupta P, McIntire SL (2002). Regulation of body size and behavioral state of C. elegans by sensory perception and the EGL-4 cGMP-dependent protein kinase.. Neuron.

[15] You YJ, Kim J, Raizen DM, Avery L (2008). Insulin, cGMP, and TGF-beta signals regulate food intake and quiescence in C. elegans: a model for satiety.. Cell Metab.

[16] Hong RL, Witte H, Sommer RJ (2008). Natural variation in Pristionchus pacificus insect pheromone attraction involves the protein kinase EGL-4.. Proc Natl Acad Sci U S A.

[17] Osborne KA, Robichon A, Burgess E, Butland S, Shaw RA (1997). Natural behavior polymorphism due to a cGMP-dependent protein kinase of Drosophila.. Science.

[18] Ben-Shahar Y, Robichon A, Sokolowski MB, Robinson GE (2002). Influence of gene action across different time scales on behavior.. Science.

[19] Hong RL, Svatos A, Herrmann M, Sommer RJ (2008). Species-specific recognition of beetle cues by the nematode Pristionchus maupasi.. Evol Dev.

[20] Poindexter (1981). The C. crescentuss: Ubiquitous Unusual Bacteria.. Microbiological Reviews.

[21] Foster, Woodruff (1946). Bacillin, A New Antibiotic Substance From a Soil Isolate of Bacillus subtilius.. J Bacteriol.

[22] Sargent (1975). Control of Cell Length in Bacillus.. J Bacteriol.

[23] Li G, Tang JX (2006). Low flagellar motor torque and high swimming efficiency of Caulobacter crescentus swarmer cells.. Biophys J.

[24] Quon KC, Marczynski GT, Shapiro L (1996). Cell Cycle Control by an Essential Bacterial Two-Component Signal Transduction Protein.. Cell.

[25] Raizen DM, Cullison KM, Pack AI, Sundaram MV (2006). A novel gain-of-function mutant of the cyclic GMP-dependent protein kinase egl-4 affects multiple physiological processes in Caenorhabditis elegans.. Genetics.

[26] Nakano Y, Nagamatsu Y, Ohshima Y (2004). cGMP and a germ-line signal control body size in C-elegans through cGMP-dependent protein kinase EGL-4.. Genes to Cells.

[27] Brooks KK, Liang B, Watts JL (2009). The influence of bacterial diet on fat storage in C. elegans.. PLoS ONE.

[28] Felix, Braendle (2010). The natural history of Caenorhbditis elegans.. Current Biology.

[29] Fitzpatrick MJ, Sokolowski MB (2004). In search of food: Exploring the evolutionary link between cGMP-dependent protein kinase (PKG) and behaviour.. Integrative and Comparative Biology.

[30] Avery L (2010). Caenorhabditis elegans behavioral genetics: where are the knobs?. BMC Biol.

[31] Hirose T, Nakano Y, Nagamatsu Y, Misumi T, Ohta H (2003). Cyclic GMP-dependent protein kinase EGL-4 controls body size and lifespan in C. elegans.. Development.

[32] Hao Y, Xu N, Box AC, Schaefer L, Kannan K (2011). Nuclear cGMP-Dependent Kinase Regulates Gene Expression via Activity-Dependent Recruitment of a Conserved Histone Deacetylase Complex.. PLoS Genet.

[33] Rae R, Riebesell M, Dinkelacker I, Wang Q, Herrmann M (2008). Isolation of naturally associated bacteria of necromenic Pristionchus nematodes and fitness consequences.. J Exp Biol.

[34] Rae R, Iatsenko I, Witte H, Sommer RJ (2010). A subset of naturally isolated Bacillus strains show extreme virulence to the free-living nematodes Caenorhabditis elegans and Pristionchus pacificus.. Environ Microbiol.

[35] Schulte RD, Hasert B, Makus C, Michiels NK, Schulenburg H (2011). Increased responsiveness in feeding behaviour of Caenorhabditis elegans after experimental coevolution with its microparasite Bacillus thuringiensis. Biol Lett.. PMID.

[36] Cronin CJ, Mendel JE, Mukhtar S, Kim YM, Stirbl RC (2005). An automated system for measuring parameters of nematode sinusoidal movement.. BMC Genet.

[37] Karbowski J, Cronin CJ, Seah A, Mendel JE, Cleary D (2006). Conservation rules, their breakdown, and optimality in Caenorhabditis sinusoidal locomotion.. J Theor Biol.

[38] Trent C, Tsung N, Horvitz HR (1983). Egg-laying defective mutants of the nematode Caenorhabditis elegans.. Genetics.

[39] O’Rourke EJ, Soukas AA, Carr CE, Ruvkun G (2009). C. elegans major fats are stored in vesicles distinct from lysosome-related organelles.. Cell Metabolism.

[40] Shtonda BB, Avery L (2006). Dietary food choice behavior in Caenorhabditis elegans.. J Exp Biol.

